# Intraocular pressure spikes after intravitreal injection and their impact on OCT angiography

**DOI:** 10.3389/fnins.2026.1819373

**Published:** 2026-07-23

**Authors:** Anthia Papazoglou, Alex Casanova, Markus Tschopp, Marcel Nico Menke

**Affiliations:** 1 Ophthalmology Department, Cantonal Hospital of Aarau, Aargau, Switzerland; 2 Ophthalmology Department, University Hospital of Zurich, Zurich, Switzerland; 3 Medical Faculty, University of Zurich (UZH), Zurich, Switzerland; 4 Ophthalmology Department, Cantonal Hospital of Lugano, Lugano, Switzerland; 5 Medical Faculty, University of Bern, Bern, Switzerland; 6 Ophthalmology Department, Inselspital Bern, Bern, Switzerland

**Keywords:** intra ocular pressure, IVI, IVT, OCTA, perfusion, quality, signal strength index, superficial capillary plexus

## Abstract

**Purpose:**

To evaluate whether acute intraocular pressure (IOP) elevation after intravitreal anti-VEGF injection affects superficial capillary plexus (SCP) perfusion metrics on optical coherence tomography angiography (OCTA).

**Methods:**

In this prospective single-center study, adults scheduled for unilateral intravitreal anti-VEGF treatment for macular edema were enrolled. Assessments were performed pre-injection (T1), early post-injection (T2), and 10 min after T2 (T3). OCTA (CIRRUS HD-OCT 5000; AngioPlex Metrix v11.0) used 6 × 6 mm fovea-centered scans. SCP vessel density (VD) and perfusion density (PD) were quantified for central (1 mm), inner (3 mm), and full macular fields (ETDRS grid). IOP was measured at T1–T3. Scans with signal strength index (SSI) < 6 or relevant motion/segmentation artefacts were excluded. Spearman correlation and multivariable regression analysis assessed associations of VD/PD with IOP and SSI.

**Results:**

Of 81 recruited subjects, 54 eyes (66.7%) finally met OCTA quality criteria at all timepoints and were analyzed. Mean IOP increased from 13.98 ± 3.09 mmHg (T1) to 31.15 ± 9.65 mmHg (T2; *p* < 0.0001) and decreased to 16.30 ± 4.60 mmHg at T3 (*p* = 0.6246 vs. T1). Full-field SCP VD and PD decreased from 15.07 ± 3.01 and 36.23 ± 7.60 (T1) to 13.76 ± 3.50 and 33.21 ± 9.08 (T2), and to 13.22 ± 2.99 and 31.73 ± 7.67 (T3). Between T1 and T2, no OCTA parameter changed significantly. At T3, all SCP parameters were significantly lower versus at T1 except for central VD (*p* = 0.1495) and central PD (*p* = 0.2894). SSI declined from 8.35 ± 1.39 (T1) to 7.72 ± 1.42 (T2; *p* = 0.0105) and 7.09 ± 1.38 (T3; *p* < 0.0001). Correlations between VD/PD and IOP were weak (r_s_ ≤ 0.17), whereas correlations with SSI were moderate-to-strong (r_s_ = 0.53–0.83).

**Conclusion:**

Intravitreal anti-VEGF injection caused a marked acute IOP spike, but most SCP OCTA metrics did not change at T2. The decline in SCP metrics at T3 coincided with reduced SSI and correlated more strongly with image quality than with IOP, indicating that post-procedural OCTA image quality may influence measured perfusion metrics; however, in the absence of blood pressure measurements, a physiological contribution cannot be excluded.

## Introduction

Vascular endothelial growth factor (VEGF) inhibitors were introduced into ophthalmology as intravitreal therapy (IVT) in 2004 and have since become a cornerstone in the management of retinal diseases, including neovascular age-related macular degeneration (nAMD), diabetic macular edema (DMO), cystoid macular edema (CMO) due to retinal vein occlusion (RVO), and secondary choroidal neovascularization (CNV) such as in degenerative myopia (Cornel et al., 2015; Fiebai and Odogu, 2017).

The clinical impact of anti-VEGF therapy has been profound, frequently not only preventing further vision loss, but in many cases, improving visual acuity (Rosenfeld et al., 2006; Nguyen et al., 2012). Nevertheless, IVT has adverse effects, one of which is a transient elevation in intraocular pressure (IOP) (Levin et al., 2021; Yamamoto et al., 2025). IOP fluctuations after IVT appear to be multifactorial, and their etiology is not yet fully clarified (Sharei et al., 2010). Two main mechanisms have been proposed: first, the additional volume injected into the vitreous cavity may induce an acute IOP spike due to a transient increase in intraocular volume (O’Bryhim et al., 2020; El Chehab et al., 2016); second, the procedure may cause anterior displacement of the lens–iris diaphragm, potentially increasing iris convexity, narrowing the anterior chamber angle, and leading to a transient component of angle closure with subsequent IOP elevation (Wen et al., 2016; Gumus et al., 2022; Jeong et al., 2017).

Given the transient nature of these post-injection IOP elevations, they are often considered clinically benign, and in this respect IVT is regarded as long-term safe (Kim et al., 2008; Falavarjani and Nguyen, 2013; Hoguet et al., 2019). However, more evidence raises concern that acute, intermittent IOP elevations may compromise retinal microvascular perfusion (Barash et al., 2020) and that perfusion variability could predispose ocular tissues to ischemic injury (Flammer et al., 2002; Choi and Kook, 2015). Further, IOP spikes have been associated with reductions in perfusion density (PD) in optical coherence tomography angiography (OCTA) imaging (Barash et al., 2020; Li et al., 2023). OCTA is a relatively recent non-invasive innovation allowing visualization of the vascular networks in living subjects by analyzing the dynamic signals generated in consecutive B-scan by moving erythrocytes in perfused blood vessels (Kashani et al., 2017; de Carlo et al., 2015; Choi et al., 2021; Chen et al., 2021; Knecht et al., 2009) while the surrounding retinal tissue is assumed to be static. On the other hand, there are only a limited number of studies assessing intra-retinal capillary perfusion in relation to IOP fluctuations (Choi et al., 2021; Chen et al., 2021). Therefore, the effect of IVTs on retinal perfusion remains uncertain.

In this study, we used OCTA to quantify changes in perfusion density (PD) and vessel density (VD) in patients receiving anti-VEGF IVT for macular edema, and we examined whether these changes were associated with post-injection IOP spikes and/or variations in image quality (signal strength index, SSI).

## Methods

### Study design and setting

Ethical approval for this prospective single-center study was granted by the Ethics Committee Northwest and Central Switzerland (EKNZ; approval ID: 2019–00587), and written informed consent was obtained from all participants. The study adhered to the tenets of the Declaration of Helsinki and complied with applicable national legal and regulatory requirements. The primary endpoint of the study was the change in superficial capillary plexus (SCP) PD and VD from pre-injection baseline (T1) to early after injection (T2). Secondary analyses included changes between T1 and T3, with T3 measured 10 min after T2.

### Participants and eligibility

Patients receiving routine anti-VEGF IVT at the Cantonal Hospital of Aarau, Switzerland were prospectively enrolled between 2018 and 2023. Eligible participants were adults (≥18 years) receiving unilateral IVT for nAMD, DMO, CMO due to RVO, or secondary CNV, who were able to fixate and to provide written informed consent. Exclusion criteria included uncontrolled ocular hypertension (baseline IOP > 25 mmHg); active uveitis or infection; media opacity precluding reliable imaging; poor fixation or nystagmus; systemic instability that could alter ocular perfusion (e.g., hypertensive crisis); pregnancy; and breastfeeding. Final eligibility required acceptable OCTA image quality (no clinically relevant motion or segmentation artefacts) and SSI ≥ 6 at all study timepoints (T1–T3) which led to further exclusions.

### Intervention day

Assessments were conducted at three timepoints: pre-injection (T1), early post-injection (T2), and 10 min after T2 (T3). At all timepoints OCTA was performed (CIRRUS HD-OCT 5000, Carl Zeiss Meditec, Dublin, USA) and IOP was measured using an iCare tonometer (iCare Finland Oy, Vantaa, Finland).

**Figure 1:**
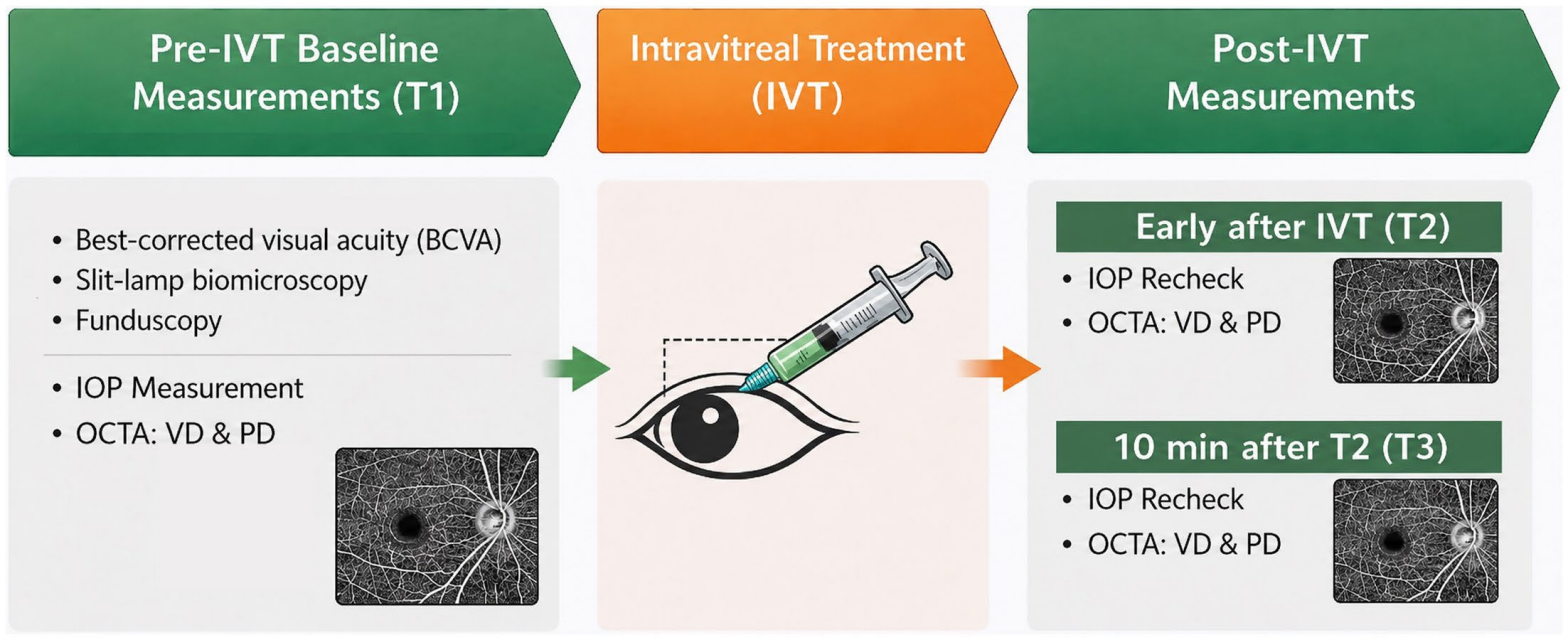
Study timeline and measurements at baseline (T1), early post-injection (T2), and 10 min after T2 (T3).

At baseline (T1), all participants underwent a standard ophthalmic examination, including best-corrected visual acuity (BCVA), slit-lamp biomicroscopy, fundus examination, IOP measurement, and OCTA of the macular and perimacular retina. At T2 and T3, IOP was re-measured and OCTA was repeated to reassess VD and PD. Systemic blood pressure (BP) was not measured at any timepoint; ocular perfusion pressure (OPP) could therefore not be estimated (see Figure 1).

### Intravitreal injection procedure

As described in previous studies, a straight IVT was performed under aseptic conditions (Knecht et al., 2009). After sterile draping, topical anesthesia with tetracaine 1% (Tetracaine 1% SDU Faure, Théa Pharma S. A., Schaffhausen, Switzerland) was administered. The periocular area was disinfected using povidone–iodine, a sterile lid speculum was inserted, and the conjunctival sac was treated with 5% povidone–iodine solution and subsequently rinsed with balanced salt solution (BSS). A 30-gauge needle was introduced through the pars plana, 3.5–4.0 mm posterior to the temporal limbus, usually in the superotemporal quadrant, to deliver 0.05 mL of medication into the vitreous cavity. Hand-movement vision was confirmed immediately after the procedure before removing the speculum. IVT agent selection was based on the treating physician’s routine clinical decision-making; the injection volume was standardized (0.05 mL) across agents.

### OCTA acquisition and analysis

Within each participant, OCTA at T1, T2, and T3 was acquired by the same physician using a standardized protocol, and repeat scans were performed when artifacts were present, to obtain the best possible image quality. Fovea-centered 6 × 6 mm OCTA scans were acquired and analyzed with AngioPlex Metrix (v11.0). Automatic quantification of PD and VD was obtained for the central 1 mm ring (central), parafoveal 3 mm (inner), and full macular fields (full) using the Early Treatment Diabetic Retinopathy Study (ETDRS) grid. As previously described, PD was defined as the fraction of the region occupied by flow signal, i.e., perfused vascular area divided by total area (%), while VD was calculated as the cumulative length of flow-positive vessels within the region of interest, normalized to the analyzed area (mm/mm^2^) (Papazoglou et al., 2026). Quantitative analyses were performed for the SCP (Li et al., 2020). After completion of patient recruitment, data analysis was initiated. All acquired OCTA scans were reviewed, and cases with insufficient OCTA image quality, defined as SSI < 6 or clinically relevant motion or segmentation artefacts at baseline or at any post-injection time point, were excluded from the analysis.

### Statistical analysis

Statistical analysis was performed using GraphPad Prism v10.4.0. Continuous variables are reported as mean ± standard deviation (SD) and categorical variables as n (%). As several variables did not meet normality assumptions (Shapiro–Wilk test), non-parametric tests were used. Repeated-measures comparisons across timepoints were performed using the Friedman test, followed by Dunn’s post-hoc pairwise comparisons with multiplicity correction for matched samples. Significance was set at *p* < 0.05. To account for multiplicity across OCTA perfusion outcomes, *p* values for the six OCTA endpoints — central, inner, and full-field VD and PD — were additionally adjusted using the two-stage linear step-up false-discovery-rate (FDR) procedure of Benjamini, Krieger and Yekutieli, with a desired false discovery rate of 5%. Correction was applied separately for each time-point. IOP and OCTA image quality/SSI were analyzed separately and were not included in this FDR correction, as they were considered physiological and image-quality variables rather than OCTA perfusion outcomes.

Spearman’s rank correlation coefficient (r_s_) was used to explore associations between pooled percentage changes from baseline in OCTA perfusion metrics (PD/VD) and percentage changes from baseline in IOP and OCTA image quality (*n* = 108 observations each). *p* values for the six OCTA endpoints were FDR adjusted separately for the IOP and SSI correlation analyses.

To further assess whether changes in IOP were independently associated with OCTA-derived perfusion metrics after accounting for image quality, exploratory multivariable linear regression analyses were performed. Percentage change from baseline in each OCTA metric was used as the dependent variable. Percentage changes in IOP and OCTA-SSI were entered simultaneously as independent variables. T2-T1 and T3-T1 were analyzed separately. Separate models were fitted for central, inner, and full-field PD and VD. Multicollinearity was assessed using variance inflation factors. *p* values were FDR adjusted.

To assess potential selection bias, baseline characteristics were compared between eyes included in the final analysis and eyes excluded from the final analysis data. Continuous variables were compared using two-tailed Mann–Whitney U tests. “Diagnosis” was treated as a nominal categorical variable and analyzed using Fisher’s exact test. p values were FDR adjusted across the four baseline comparisons.

## Results

**Figure 2:**
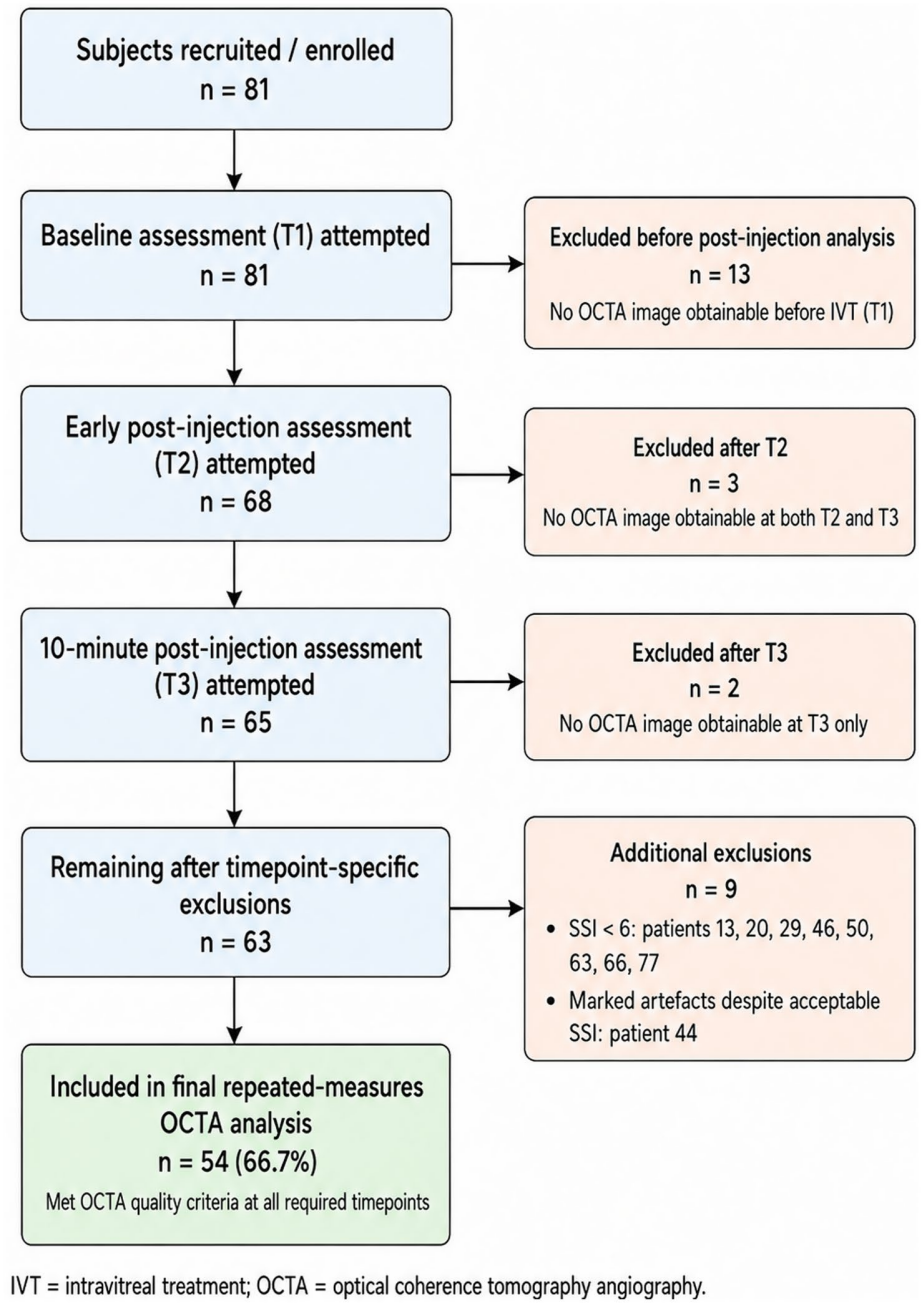
Participant flow with OCTA image-quality exclusions at post-injection timepoints.

An initial total of 81 subjects were recruited. Of these, 54 eyes (66.7%) with OCTA scans of sufficient quality at baseline (T1) and at both post-injection time points (T2, T3) were included in the final analysis. As depicted in the flowchart (Figure 2), 27 subjects were excluded due to insufficient image quality and/or artefacts. Included and excluded eyes did not differ significantly with respect to age, baseline BCVA, pre-injection IOP, or the diagnosis (see Table S1). OCTA acquisition at T2 commenced typically within 7.5 min (median) and T3 7–15 min after T2. The mean age of the included patients was 75.31 ± 9.68 years, and 30 participants were female. Right and left eyes were similarly represented (27 and 27 eyes, respectively). The most frequent treatment indication was nAMD, followed by DMO and CNV (36,13,4). The injected agents administered according to routine clinical practice were aflibercept (Eylea) 23/54 (42.6%), ranibizumab (Lucentis) 22/54 (40.7%), and faricimab (Vabysmo) 9/54 (16.7%). All patients’ treatment status was ongoing; no naïve patients were included.

### Intraocular pressure

Mean IOP was 13.98 ± 3.09 mmHg at T1 and increased significantly to 31.15 ± 9.65 mmHg at T2 (*p* < 0.0001). IOP subsequently decreased to 16.30 ± 4.60 mmHg at T3, which was not significantly different to the IOP at baseline T1 (*p* = 0.6246).

### OCTA parameters (SCP vessel density and perfusion density)

**Figure 3:**
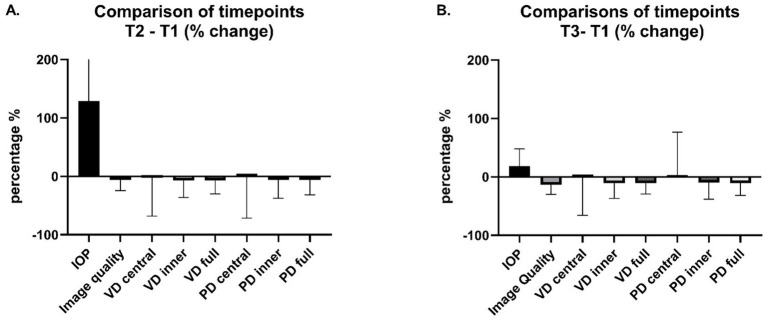
**(A)** Relative percentage change between baseline (T1) and early after intravitreal injection (T2) in intraocular pressure (IOP), OCTA image quality, vessel density (VD), and perfusion density (PD). **(B)** Relative percentage change between baseline (T1) and 10 min after T2 (T3) in intraocular pressure (IOP), OCTA image quality, vessel density (VD), and perfusion density (PD). Quantitative OCTA parameters are shown for the central, inner, and full macular regions. Bars represent mean percentage change, and error bars indicate standard deviation.

Full-field mean PD and VD decreased from 15.07 ± 3.01 and 36.23 ± 7.60 at T1 to 13.76 ± 3.50 and 33.21 ± 9.08 at T2. They continued declining even further to 13.22 ± 2.99 and 31.73 ± 7.67 at T3. None of the OCTA parameters showed a statistically significant change between T1 and T2. When comparing T1 with T3, all quantitative OCTA parameters showed a statistically significant decrease, with the exception of central mean PD (*p* = 0.2894) and VD (*p* = 0.1495). Central, inner, and full mean PD and VD values are displayed in Table 1 and Figure 3.

**Table 1:** Changes in IOP, OCTA image quality, and macular OCTA perfusion metrics across study timepoints (*n* = 54).

**Changes in IOP and macular OCTA metrics**		Study timeline						
		**T1**	**T2**	*p*-value **(T2 vs T1)**	**T3**	*p*-value **(T3 vs T1)**	**Change T2-T1**	**Change T3-T1**
	OCTA image quality (SSI)	8.35 (1.39)	7.72 (1.42)	0.0105	7.09 (1.38)	<0.0001	−5.97% (18.33)	−13.35% (16.85)
	IOP (mmHg)	13.98 (3.09)	31.15 (9.65)	<0.0001	16.30 (4.60)	0.6246	128.64% (76.15)	18.29% (29.46)
	VD (mm/mm²), central	8.27 (4.68)	7.21 (4.80)	0.1131	7.17 (4.82)	0.1495	−2.59 (65.55)	−0.59 (65.39)
	VD (mm/mm²), inner	14.59 (3.58)	13.24 (4.35)	0.1527	12.72 (3.69)	0.0003	−6.64 (29.60)	−10.80 (26.18)
	VD (mm/mm²), full	15.07 (3.01)	13.76 (3.50)	0.1130	13.22 (2.99)	0.0002	−6.71 (23.50)	−10.91 (18.56)
	PD (%), central	18.23 (11.02)	15.86 (11.17)	0.1130	15.97 (11.53)	0.2894	−0.07 (71.47)	3.23 (73.44)
	PD (%), inner	34.41 (8.82)	31.33 (10.99)	0.3127	30.14 (9.00)	0.0064	−5.83 (31.68)	−9.42 (28.59)
	PD (%), full	36.23 (7.60)	33.21 (9.08)	0.1527	31.73 (7.67)	0.0005	−6.06 (25.55)	−10.70 (21.02)

All values are presented as mean (SD). *p*-values represent pairwise comparisons versus baseline (T1) and were obtained using Dunn’s multiple-comparisons test following an overall Friedman test for repeated measures. False discovery rate (FDR) correction for *p*-values was applied to OCTA perfusion endpoints separately for the T2–T1 and T3–T1 time-points. “Change (%)” indicates the percentage change from baseline (mean SD).

IOP, intraocular pressure; OCTA, optical coherence tomography angiography; VD, vessel density; PD, perfusion density.

### OCTA image quality (signal strength index - SSI)

SSI was 8.35 ± 1.39 at baseline (T1), decreased to 7.72 ± 1.42 at T2, and declined further to 7.09 ± 1.38 at T3. The reduction in SSI was statistically significant between T1 and T2 (*p* = 0.0105) and between T1 and T3 (*p* < 0.0001).

### Correlation analysis between OCTA perfusion metrics (VD/PD), IOP, and OCTA image quality

Spearman correlation analyses were performed to assess the relationship between percentage changes from baseline in OCTA-derived perfusion metrics, IOP, and OCTA image quality (SSI). Percentage changes were analysed with pooled data from both post-injection intervals.

Changes in PD and VD showed no significant association with changes in IOP (r_s_ range, −0.030 to 0.173; all *p* ≥ 0.1785) (Table 2; Figure S1). In contrast, these showed moderate-to-strong positive associations with changes in SSI (r_s_ range, 0.566–0.829; all p < 0.0001) (Table 2; Figure S1). In exploratory multivariable regression models adjusted for SSI, percentage change in SSI remained independently associated with percentage changes in all OCTA metrics across all analyzed regions and at both post-injection intervals (T2–T1: *β* range, 0.965–1.830; adjusted p < 0.0001–0.0012; T3–T1: β range, 0.833–2.613; all adjusted p < 0.0001; Table S2). By contrast, percentage change in IOP was not independently associated with any OCTA-derived VD or PD metric after adjustment for SSI at either interval (T2–T1: β range, 0.00006–0.082; adjusted *p* = 0.945–1.000; T3–T1: β range, −0.140 to −0.025; adjusted p = 0.945–0.960).

**Table 2:** Spearman correlations of changes in OCTA perfusion metrics with IOP and OCTA image quality (*n* = 108).

**Changes in macular OCTA perfusion metrics**		**Changes in intraocular pressure**			**Changes in OCTA image quality**		
		**IOP** r_s_	**95% confidence interval**	*p*-value	**OCTA image quality r_s_**	**95% confidence interval**	*p*-value
	**VD (mm/mm²) central**	−0.0301	−0.2232 to 0.1653	0.8014	0.5657	0.4172 to 0.6848	<0.0001
	**VD (mm/mm²) inner**	0.1717	−0.0236 to 0.3543	0.1785	0.7471	0.6466 to 0.8221	<0.0001
	**VD (mm/mm²) full**	0.1733	−0.0218 to 0.3558	0.1785	0.8207	0.7452 to 0.8755	<0.0001
	**PD (%) central**	−0.029	−0.2225 to 0.1660	0.8014	0.5859	0.4418 to 0.7005	<0.0001
	**PD (%) inner**	0.1396	−0.0564 to 0.3252	0.2356	0.7577	0.6606 to 0.8299	<0.0001
	**PD (%) full**	0.1665	−0.0288 to 0.3496	0.1785	0.8289	0.7563 to 0.8813	<0.0001

Spearman’s rank correlation coefficient (r_s_) was used to assess the association between OCTA perfusion metrics (VD/PD) and IOP as well as OCTA image quality. P- values are false discovery rate (FDR) corrected for IOP and OCTA image quality.

IO, intraocular pressure; OCTA, optical coherence tomography angiography; VD, vessel density; PD, perfusion density.

## Discussion

The association between elevated IOP and reduced retinal perfusion has been the subject of various experimental and clinical studies. In the present study, we examined the effect of acute IOP elevation on macular perfusion in eyes undergoing anti-VEGF IVT using OCTA. Consistent with previous reports focused either on the macular region (Barash et al., 2020; Li et al., 2023; Arumuganathan et al., 2021) or on the peripapillary/optic nerve head region (Wen et al., 2016; Viggiano et al., 2024), our study confirmed that an IOP spike occurs early after IVT. IOP increase observed in our cohort was within the range of previously reported post-IVT increases, exceeding those reported by Arumuganathan et al. (2021) and Viggiano et al. (2024), but remaining below that reported by Wen et al. (2016). However, these cross-study comparisons should be interpreted with caution, as the timing of the first post-interventional measurements varied substantially, and therefore likely captured different phases of the IOP peak and recovery. This variability in acquisition timing may also contribute to differences in reported OCTA findings across studies. For example, Barash et al., 2020 reported perfusion density reductions after “immediate” post-IVT imaging, whereas Arumuganathan et al. (2021) with measurements acquired 5 min post-intervention, did not observe statistically significant cohort-level quantitative changes. The present study aligns more closely with Arumuganathan et al. (2021), as the T2 measurements were acquired approximately 7 min after the IVT and no statistically significant decrease in macular SCP PD or VD.

An overall decrease in most quantitative SCP OCTA parameters was noted across the wider interval from T1 to T3. This could reflect a physiological effect, such as IOP-related perfusion changes and/or a technical effect related to reduced image quality. To explore these possibilities, Spearman correlation analyses were performed to assess associations between changes in PD/VD and changes in IOP, as well as between changes in PD/VD and SSI. The correlation between PD/VD and IOP was weak (r_s_ ≈ 0.17 or lower), whereas between PD/VD and SSI was moderate to strong (r_s_ = 0.53–0.83; Table 2). These findings suggest no meaningful association between OCTA-derived perfusion metrics and IOP in this cohort, but a substantial association with image quality. Exploratory multivariable regression analyses further supported this pattern: percentage change in SSI remained independently associated with all VD and PD metrics at both post-injection time points, whereas percentage change in IOP was not independently associated with any OCTA metric after adjustment for SSI (Table S2). Taken together, these analyses suggest that the observed decline in VD/PD, particularly at T3, may largely reflect reduced image quality rather than a direct physiological reduction in retinal perfusion caused by IOP elevation.

This interpretation is consistent with prior evidence suggesting that acute IOP changes alone may not fully explain the variability of OCTA-derived perfusion metrics after IVT. Li et al. (2023) reported a superficial macular VD reduction 30 min post-intervention, when IOP had largely normalized, indicating that factors other than IOP may influence measured perfusion parameters. These factors may include measurement noise, segmentation stability, autoregulatory responses, and scan quality. Similarly, Wen et al. (2016). observed significant perfusion changes in later early post-injection acquisitions rather than the earliest acquisition. While this may suggest that measurable perfusion effects can evolve over time, acquisition-dependent factors, including SSI, motion artefacts, and segmentation variability, may also contribute during the immediate post-interventional period. Arumuganathan et al. (2021) and Viggiano et al. (2024) did not detect an overall statistically significant perfusion density change attributable to IVT.

Despite its prospective design, this study has limitations. First, BP was not measured at T1, T2, or T3; therefore, OPP could not be estimated. In clinical research, OPP is commonly approximated as the difference between BP (often expressed as two-thirds of the mean arterial pressure to account for eye-level pressure in the upright position), and IOP (Leske, 2009). This is relevant because IVT may induce stress or anxiety, with a transient increase in BP (Berger et al., 2019). In the FEAR study, BP increased during the injection procedure, but decreased again by the post-injection measurement approximately 5 ± 2 min later; according to the presented graphical assessment, values appeared closer to baseline than to the intra-injection peak (Berger et al., 2019). This suggests that procedure-related BP elevation may be less pronounced during early post-injection assessments than during the injection itself. Nevertheless, a compensatory increase in BP could theoretically attenuate the reduction in OPP caused by the acute IOP spike and thereby mask a true reduction in retinal perfusion on OCTA (Figueiredo et al., 2013). Consequently, our study cannot distinguish between true stability of retinal perfusion and preserved OCTA metrics due to systemic hemodynamic compensation. Future studies should incorporate concurrent BP measurements to enable OPP estimation and improve physiological interpretability of early post-injection OCTA changes. Second, IOP was not measured in the contralateral non-injected eye. Concurrent fellow-eye measurements could have served as an internal control to better differentiate injection-related IOP elevation from systemic or procedure-related influences (e.g., stress response, posture, lid squeezing) that may affect IOP measurements. Third, the exclusion rate was high with 27 of 81 patients excluded because the predefined quality criteria were not met at all timepoints. Reduced OCTA image quality after intravitreal procedures is a recognized challenge in the literature (Papazoglou et al., 2026) and is commonly attributed to ocular surface dryness and debris following povidone-iodine exposure and prolonged speculum time. However, this may introduce selection bias and limits generalizability to the broader population of patients undergoing intravitreal injection. Finally, only eyes with retinal pathologies were included; therefore, the extent to which intravitreal application would affect IOP and retinal perfusion in healthy eyes remains unclear.

## Conclusion

At T2, IOP increased significantly, but no significant changes were detected in quantitative OCTA parameters of the superficial capillary plexus. These findings suggest that the acute IOP rise was not associated with a detectable reduction in macular retinal perfusion within the early post-injection interval examined.

At T3 (10 min after T2), changes in OCTA metrics coincided with reduced image quality and were more strongly associated with SSI than with IOP, suggesting that post-procedural image quality may substantially influence measured perfusion metrics. Physiological interpretation remains limited because systemic blood pressure was not measured, and OPP could not be estimated.

## Data Availability

The raw data supporting the conclusions of this article will be made available by the authors, without undue reservation.
